# Editorial: Inflammasomes in human diseases and metabolism

**DOI:** 10.3389/fimmu.2024.1420303

**Published:** 2024-05-22

**Authors:** Gabriel Mbalaviele, Bernhard Ryffel

**Affiliations:** ^1^Division of Bone and Mineral Diseases, Department of Medicine, School of Medicine, Washington University, St. Louis, MO, United States; ^2^INEM, CNRS, UMR7355, Université d’Orléans, Orléans, France

**Keywords:** inflammation, metabolism, inflammasome, pyroptosis, NLRP3

## Introduction

Inflammasomes are intracellular multiprotein complexes that are assembled upon cell recognition or sensing of pathogen- or danger-associated molecular patterns (1–4). They are responsible for the maturation of IL-1β and IL-18 and can cause the inflammatory cell death, pyroptosis (5, 6). Dysregulated inflammasome activities are implicated in a variety of pathologies, including metabolic diseases such as obesity, diabetes, gout, and atherosclerosis (7–14). This Research Topic focuses on the role of these protein complexes in selected aspects of inflammation and metabolic diseases, with the purpose of providing mechanistic insights that can be leveraged for the development of novel therapeutic modalities.

The NOD-like receptor family pyrin domain containing 6 (NLRP6)-nucleated inflammasome) is involved in intestinal inflammation. Nascimento et al. report a role for this inflammasome in chronic obstructive pulmonary disease (COPD) caused by cigarette smoke (CS) (Nascimento et al., 2023). Wild-type (WT) mice exposed to CS develop airway inflammation, a response that is mediated by lung epithelial cells, which are severely impaired in mice lacking *Nlrp6*. Since gut-derived metabolites regulate NLRP6 inflammasome activation in intestinal epithelial cells, the authors investigate the link between NLRP6, CS-driven lung inflammation, and gut microbiota composition. They find that CS exposure disrupts gut microbiota in both WT and *Nlrp6-deficient* mice; the transfer of intestinal microbiota from dysbiotic *Nlrp6*-deficient mice to their WT counterparts reduces lung inflammation, highlighting a NLRP6-dependent gut-to-lung axis that controls pulmonary inflammation.

Different inflammasomes are known to be activated in neurodegenerative diseases, such as amyotrophic lateral sclerosis (ALS). To better understand the role that the NLRP3 inflammasome plays in ALS, Clénet et al. pharmacologically inhibit this pathway in relevant cell systems and the SOD1^G93A^ ALS experimental model (Clenet et al., 2023). They find that inhibition of the NLRP3 inflammasome fails to prevent disease progression, an outcome that may be due to functional redundancy among inflammasome pathways.

Evidence suggests that gut microbiota components or metabolites may directly or indirectly modulate NLRP3 inflammasome activation. On the one hand, an association between cardiac atrial fibrillation (AF) and NLRP3 activation has been reported. On the other hand, intestinal dysbiosis has been associated with the development of AF. This review analyzes the interconnection of NLRP3 inflammasome and intestinal dysbiosis during the onset and persistence of AF. Furthermore, the potential value of pharmacological and dietary changes for the prevention or treatment of AF is discussed (Xing et al., 2023).

A study by Lou et al. investigates gut dysbiosis in experimental sepsis in mice and humans (15). Antibiotic therapy can reduce patients’ commensal bacterial population and increase their risk of developing subsequent diseases. The authors report that the composition of the gut microbiota differs significantly between healthy patients and those with sepsis. At the phylum level, the amount of Proteobacteria in the intestinal flora of patients with sepsis is much higher than that of the control group, while the number of Firmicutes is significantly lower. Mice with gut microbiota disorders are found to have an increased risk of death, inflammation, and organ failure as compared to mice subjected to cecal ligation and puncture. Fecal microbial transplantation (FMT) and short-chain fatty acids (SCFAs) regulate the abundance of bacteria such as Firmicutes, Proteobacteria, Escherichia, Shigella, and Lactobacillus, restoring them to levels comparable to those of healthy mice. Furthermore, FMT and SCFAs increase the expression of the tight junction protein occludin in the colon of mice with sepsis, downregulate the expression of NLRP3 and GSDMD, and reduce the release of cytokines (e.g., IL-1β and IL-18) and pyroptosis. In conclusion, FMT and SCFAs provide a survival benefit in a mouse model of sepsis and may be a viable treatment for sepsis in humans.

Although the majority of published data relate to the NLRP3 inflammasome in immune cells, activation of this pathway in parenchymal cells, namely epithelial cells, has also been published. Indeed, activation of this inflammasome in podocytes and epithelial cells releases IL-β and is associated with barrier dysfunction. Using single-cell transcriptome analysis, Kunte et al. find no NLRP3 expression in parenchymal cells of the kidney, and no response of primary human podocytes to NLRP3 agonists (Kunte et al., 2023). The authors also find that diabetes-induced glomerulopathy is indistinguishable between WT mice and mutant mice expressing constitutively activated NLRP3 in podocytes. Intriguingly, the disease is reversed upon feeding mice with the NLRP3 inflammasome agonist, ß-hydroxy-butyrate. The authors conclude that NLRP3-mediated glomerular inflammation is dependent on immune cells, but not on parenchymal cells.

The link between NLRP3 inflammasome activation and metabolism is not well understood, a knowledge gap that is reviewed and discussed by Ortega et al. (Ortega et al., 2023). Understanding this interplay may lead to the design of specific inhibitors for the treatment and prevention of various immune or metabolic diseases.

## Author contributions

GM: Writing – original draft, Writing – review & editing. BR: Writing – original draft, Writing – review & editing.

## References

[1] Martinon F Burns K Tschopp J. The inflammasome: a molecular platform triggering activation of inflammatory caspases and processing of proIL-beta. Mol Cell. (2002) 10:417–26. doi: 10.1016/S1097-2765(02)00599-3 12191486

[2] Agostini L Martinon F Burns K McDermott MF Hawkins PN Tschopp J. NALP3 forms an IL-1beta-processing inflammasome with increased activity in Muckle-Wells autoinflammatory disorder. Immunity. (2004) 20:319–25. doi: 10.1016/S1074-7613(04)00046-9 15030775

[3] Petrilli V Papin S Tschopp J. The inflammasome. Curr Biol. (2005) 15:R581. doi: 10.1016/j.cub.2005.07.049 16085473

[4] Fu J Wu H. Structural mechanisms of NLRP3 inflammasome assembly and activation. Annu Rev Immunol. (2023) 41:301–16. doi: 10.1146/annurev-immunol-081022-021207 PMC10159982 36750315

[5] Broz P Pelegrin P Shao F. The gasdermins, a protein family executing cell death and inflammation. Nat Rev Immunol. (2020) 20:143–57. doi: 10.1038/s41577-019-0228-2 31690840

[6] Chen KW Demarco B Broz P. Beyond inflammasomes: emerging function of gasdermins during apoptosis and NETosis. EMBO J. (2020) 39:e103397. doi: 10.15252/embj.2019103397 31793683 PMC6960442

[7] Gasse P Mary C Guenon I Noulin N Charron S Schnyder-Candrian S. IL-1R1/MyD88 signaling and the inflammasome are essential in pulmonary inflammation and fibrosis in mice. J Clin Invest. (2007) 117:3786–99. doi: 10.1172/JCI32285 PMC2066195 17992263

[8] Gasse P Riteau N Charron S Girre S Fick L Petrilli V. Uric acid is a danger signal activating NALP3 inflammasome in lung injury inflammation and fibrosis. Am J Respir Crit Care Med. (2009) 179:903–13. doi: 10.1164/rccm.200808-1274OC 19218193

[9] Besnard AG Guillou N Tschopp J Erard F Couillin I Iwakura Y. NLRP3 inflammasome is required in murine asthma in the absence of aluminum adjuvant. Allergy. (2011) 66:1047–57. doi: 10.1111/j.1398-9995.2011.02586.x 21443539

[10] Elinav E Strowig T Kau AL Henao-Mejia J Thaiss CA Booth CJ. NLRP6 inflammasome regulates colonic microbial ecology and risk for colitis. Cell. (2011) 145:745–57. doi: 10.1016/j.cell.2011.04.022 PMC3140910 21565393

[11] Hirota SA Ng J Lueng A Khajah M Parhar K Li Y. NLRP3 inflammasome plays a key role in the regulation of intestinal homeostasis. Inflammation Bowel Dis. (2011) 17:1359–72. doi: 10.1002/ibd.21478 PMC3026862 20872834

[12] Besnard AG Togbe D Couillin I Tan Z Zheng SG Erard F. Inflammasome-IL-1-Th17 response in allergic lung inflammation. J Mol Cell Biol. (2012) 4:3–10. doi: 10.1093/jmcb/mjr042 22147847

[13] Menu P Mayor A Zhou R Tardivel A Ichijo H Mori K. ER stress activates the NLRP3 inflammasome via an UPR-independent pathway. Cell Death Dis. (2012) 3:e261. doi: 10.1038/cddis.2011.132 22278288 PMC3270266

[14] Canadas-Lozano D Marin-Aguilar F Castejon-Vega B Ryffel B Navarro-Pando JM Ruiz-Cabello J. Blockade of the NLRP3 inflammasome improves metabolic health and lifespan in obese mice. Geroscience. (2020) 42:715–25. doi: 10.1007/s11357-019-00151-6 PMC7206474 31975052

[15] Lou X Xue J Shao R Mo C Wang F Chen G. Postbiotics as potential new therapeutic agents for sepsis. Burns Trauma. (2023) 11:tkad022. doi: 10.1093/burnst/tkad022 37334140 PMC10271603

